# Detection and distribution of Sca autotransporter protein antigens in diverse isolates of *Orientia tsutsugamushi*

**DOI:** 10.1371/journal.pntd.0006784

**Published:** 2018-09-20

**Authors:** Munegowda C. Koralur, Arunachalam Ramaiah, Gregory A. Dasch

**Affiliations:** Rickettsial Zoonoses Branch, Centers for Disease Control and Prevention, Atlanta, GA, United States of America; Instituto de Pesquisas Veterinarias Desiderio Finamor, BRAZIL

## Abstract

*Orientia tsutsugamushi* (Ots) frequently causes severe scrub typhus infections in the Asia-Pacific region. Korean investigators have demonstrated that Ots encodes five different autotransporter domain (ATD) proteins (ScaA-ScaE). ScaA functions as an adhesin and confers protective immunity in a lethal mouse model of Ots infection. Specific antibodies are detected against ScaA and ScaC in Korean scrub typhus patients. However, there is limited data on the distribution of the Sca protein genes in diverse isolates of Ots. By BLAST analysis with the conserved beta barrel autotransporter domain (ATD) regions of the *sca* proteins, we discovered a sixth gene *sca*F among 3 of 10 new partial Ots genome sequences available at NCBI GenBank (Sido, Karp, AFSC7). We designed two to seven specific TaqMan assays to detect the ATD for each of the six *sca* genes. The TaqMan assays among those for each *sca* gene which gave the greatest sensitivity and linearity with DNA log dilutions were then used for screening DNAs from Ots isolates grown in L929 mouse cells for *sca* genes. The *sca* prevalence survey was performed for all six *sca* genes with 178 DNAs from isolates from 12 countries. The survey results were confirmed by conventional PCR with primers from conserved regions of the passenger domains (PD) and ATD of the *sca* proteins. The ATD was highly conserved between the DNAs of different genotypes compared to the *sca* PD but each TaqMan assay was *sca* specific. The percentage positivity for 56 kDa and *sca*A genes in the 178 DNAs using Ha primers was 59.6% and 62.4%, respectively. Our *sca*A conventional ATD PCR assay was positive in 98.3% but *sca*A was present in all 178 DNAs (100%) by ATD TaqMan. *sca*B, *sca*C, *sca*D, *sca*E and *sca*F were detected in 33.7%, 97.8%, 93.8%, 97.2% and 43.3% isolates by ATD TaqMan, respectively. The ATDs of Ots *sca* genes are thus sufficiently conserved between different genotypes for molecular assay design. Four *sca* genes are widely distributed among diverse Ots isolates from diverse geographical areas. *sca*B and *sca*F were detected in fewer Ots isolates and absent from some available genome sequences. Whether the utility of the ScaA, ScaC, ScaD, and ScaE antigenic passenger protein domains exceeds that of the mixed 56 kDa type surface antigens of Ots now used in combination diagnostic assays needs to be determined before they can be considered as suitable alternative serological antigens for diagnosis of scrub typhus.

## Author summary

*Orientia tsutsugamushi* (Ots) frequently causes severe scrub typhus infections in the Asia-Pacific region. Korean investigators had previously demonstrated that Ots encodes five different cell surface (Sca) proteins which have functional regions that mediate their transport to the cell surface. One of the proteins (ScaA) is able to serve as a vaccine against a Korean strain of Ots. Several of the Sca proteins stimulate production of antibodies during scrub typhus infections in humans. However, very little was known about the distribution of the Sca protein genes in isolates of Ots in other countries in the Asia-Pacific region where scrub tyhus occurs. We discovered there is a sixth gene *sca*F in some Ots strains. We designed sensitive molecular assays from conserved regions of each protein to survey the presence of the six *sca* genes in 178 DNAs from isolates from 12 countries. Only four *sca* genes are widely distributed among diverse Ots isolates from diverse geographical areas. *sca*B and *sca*F were detected much less frequently in Ots isolates. Future studies will be required to determine whether the Sca proteins are suitable for improved diagnostic assays and vaccines for scrub typhus.

## Introduction

Scrub typhus, caused by *Orientia tsutsugamushi* (Ots), is a common acute febrile illness in the Asia Pacific region; however, related agents with a few cases of associated disease have been described recently in new places in Africa, South America and related agents may occur in Europe [1–4]. Scrub typhus presents with fever, headache, chills, myalgia, and arthralgia, as is common with other tropical febrile illnesses, but may also present with a characteristic diagnostic eschar at the site(s) of the vector chigger bites [5] The severity of the disease can range from mild forms with low grade fever to severe fatal illnesses with high grade fever and complications involving multiple organ failure. The prevalence of disease has increased in recent years partly because of improved diagnostic methods and its recognition in many new foci; indeed, in many endemic regions it is the leading cause of treatable non malarial illness [6]. The public health impact of this disease is huge as at least a billion people are at risk of this disease with at least a million cases suspected annually [6, 7]. Appropriate and timely antibiotic treatment with doxycycline or chloramphenicol generally leads to a very good prognosis [8, 9]; however, recognizing the disease early and with certainty has been a significant public health issue, especially in the rural parts of the endemic world which lack access to specific diagnostic tests [10]. Since clinical diagnosis can be difficult due to overlapping signs and symptoms with other tropical febrile illnesses, confirmatory diagnosis is still largely based on non-specific Weil-Felix laboratory tests in many district hospitals [10].

Gram negative bacteria (GNB) including the Rickettsiales with two cell membranes (diderms) have complex cell envelopes with a cytoplasmic membrane, periplasmic space, outer membrane and frequently surface layer molecules that have roles in virulence. This presents a challenge for the transport of the surface and outer membrane proteins, lipopolysaccharides and capsule components across the cell envelope. [11]. Protein transport systems in GNB are termed as types I to IX [12]. The simplest secretion pathway was originally thought to be the intrinsic type V autotransporter (AT) secretion system which employs an N-terminal signal sequence recognized by the Sec machinery for passenger domain (PD) transport across the cytoplasmic membrane and the attached transmembrane beta barrel autotransporter domain (ATD) for outer membrane transport [13]. However, it is now recognized that beta-barrel assembly machinery (BAM) proteins and in some cases translocation and assembly module proteins (TAM) are essential for the translocation of ATD proteins [14]. The ATD proteins, many of which are cell surface proteins, often play an important role in GNB virulence functions such as adhesion, aggregation, invasion, biofilm production and toxicity and are now being exploited in bacteria for surface display of other protein moieties [15]. Among the sequenced *Rickettsia* genomes, a family of 17 ATD paralogous genes has been identified which are also called the surface cell antigen (*sca*) genes [16]. The major *sca*/ATD genes *omp*A, *omp*B, and *sca*4 are used for differentiating species of *Rickettsia* [17]. The roles in pathogenesis of five of these proteins (OmpA, OmpB, Sca1, Sca2, and Sca4 have been well studied in *Rickettsia* [16–22]. In particular, the OmpA and OmpB proteins are important antigens conferring protective immunity to infection and because they are immunodominant antigens, they are targets for serological diagnosis of infections due to *Rickettsia* [23].

In contrast to the significant amount of attention given the *sca*/ATD proteins of *Rickettsia*, the *sca*/ATD proteins of their nearest relatives in the genus *Orientia* have received scant attention. Korean investigators have demonstrated that Ots encodes five different autotransporter domain (ATD) proteins (ScaA-ScaE) [24] based on the two complete genomes of *Orientia tsutsugamushi* strains (Boryong, 2007 and Ikeda, 2008) then available [25, 26]. ScaA of Boryong strain functions as an adhesin and confers protective immunity in a lethal mouse model of Ots infection [27]. Specific antibodies were also detected against ScaA and ScaC in Korean scrub typhus patients [28]. Ha et al. (2012) detected and sequenced 4 of the 5 Sca Genes in three other prototype isolates of *Orientia* (Gilliam, Karp, Kato) by use of conserved Boryong-Ikeda derived PCR primers [28]. However, there is only limited data available on the distribution of homologues of these Sca protein genes in antigenically and genetically diverse isolates of *Orientia tsutsugamushi*.

Serological and molecular tests for scrub typhus are more commonly used than culture based assays, as these bacteria are obligately intracellular and thus require antibiotic-free cell cultures for cultivation. Furthermore, BSL-3 conditions are recommended for producing specific *Orientia* antigens and reagents; this is both costly and highly restricted to a few specialty laboratories. The most common gene target for both serological assays such as Dip-Stick, Flow Assays, and ELISA and molecular diagnosis has been the 56kDa scrub typhus type specific surface antigen (TSA) [29]. Since this gene exhibits substantial genetic variability, it is the target most commonly used for differentiation and typing of the infecting genotypes of *O*. *tsutsugamushi* [30]. However, because of this TSA antigenic diversity, matching a clinical antibody response to Ots to the particular antigenic type of Ots infecting a patient is desirable to assure high sensitivity of serological detection [31, 32]. Consequently, while serological tests with mixed STA56 antigenic types have been employed to overcome this limitation or reliance on the presence of cross-reactive antibodies to single antigen types have been used, they may be inappropriate for geographic regions whose Ots isolate types are unknown or for antigenically distant infecting strains. Another recombinant protein antigen target for serological diagnosis of scrub typhus is the more conserved STA47 antigen gene of Ots but the antibody response for this target may be delayed in comparison to 56kDA TSA, especially in acute phase sera [33]

In the present work, we have assessed the prevalence of *O*. *tsutsugamushi sca* protein genes in a large collection of isolates from the Asia-Pacific region by means of new specific TaqMan assays targeting conserved sites in each of the *sca*A-E autotransporter domains. We also describe the TaqMan detection of a new ATD protein gene, *sca*F. The assays were designed based on available *sca* gene data extracted from partial genome sequences of Ots available at NCBI in 2017 when the study was performed.

## Materials and methods

### *Orientia tsutsugamushi* genome database analysis

Using tBLASTn (search translated nucleotide databases using a protein query) [https://blast.ncbi.nlm.nih.gov/Blast.cgi?CMD=Web&PAGE_TYPE=BlastHome] with the five complete Boryong ScaA-ScaE protein sequences available from Ha et al. [24, 27, 28] we analyzed the two complete and 12 partial Ots genome sequences available in NCBI database (S1 Table) for the presence of different *sca* genes. The different *sca* protein genes we found were also classified based on their greatest protein (coverage and identity) tBLASTn sequence homologies with Boryong Sca proteins (S2 Table). An additional ScaF protein (645 aa) was detected in Sido, AFSC7, Karp2M and KarpIGS contigs and its similarity and distribution was further evaluated by tBLASTn of the genome sequences with the Karp ScaF sequence (S1 and S2 Tables). The presence and size of N-terminal putative signal sequences or transmembrane regions and ATD in these Sca protein genes was determined using SMART (simple modular architecture research tool [http://smart.embl-heidelberg.de/]). Full *sca* gene and DNA sequences adjacent to the 5’ and 3’ ends of the complete *sca* genes were extracted in order to design primers suitable for amplifying all of the PD and ATD of the *sca* genes detected in Ots. In some cases where incomplete genes or adjacent fragments of ATD were annotated, frame shift errors were corrected (generally single base insertions or deletions) and overlapping or adjacent contig sequences were joined based on the closest *sca* genes available in each class (S1 Table); .tBLASTn and BLASTn were performed again on the genomes with the closest homologous sequences available for genomes in which only partial sequences (S1 Table) were initially detected to ensure they were not overlooked.

### Sca sequence analysis and phylogenetic relationships

The *sca* protein gene regions (PD and ATD) identified for *O*. *tsutsugamushi* were aligned using Muscle [34] as embedded in Geneious R 9.1.6 [35]. The ATD regions were identified with SMART and the signal peptide plus passenger domain (SP-PD) region was defined as the complete region 5’ or N-terminal to the consensus ATD region predicted by SMART. In some cases this included clearly predicted signal sequences and in others, only transmembrane domains at the amino terminus. The number of amino acid identities and percentage amino acid identifies for SP-PD and ATD were obtained from Geneious (S2 Table).

**Fig 1:**
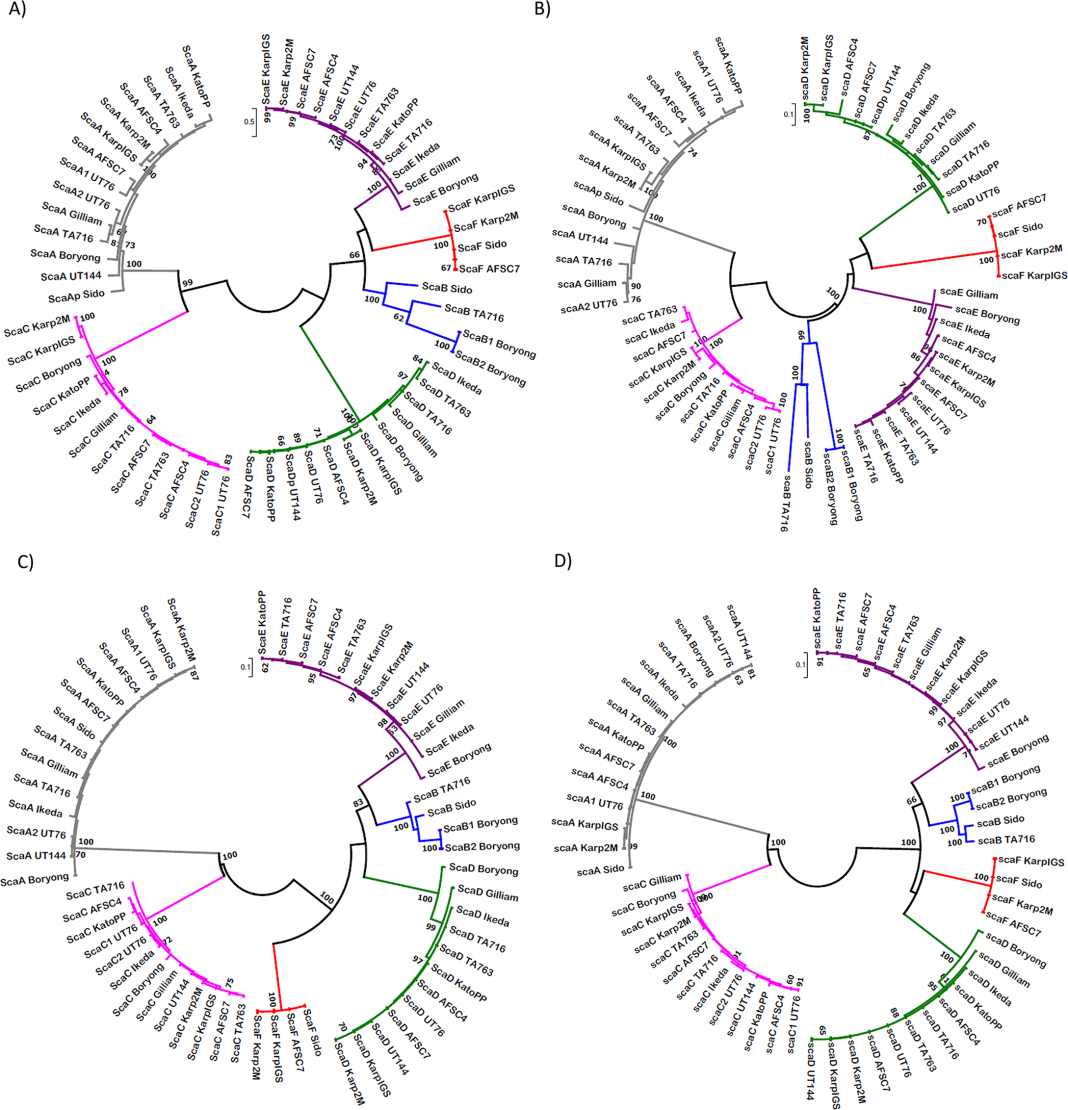
Phylogenetic relationship of *sca* protein gene sequences (types A-F) of *O*. *tsutsugamushi*. The Maximum Likelihood trees show the phylogenetic relationship of the known *sca* protein genes, based on the SP-PD region A) protein and B) gene sequences and ATD region C) protein and D) gene sequences. Bootstrap values (percentages of 100 replications) above 60% are indicated at the nodes. All four trees show two major clades, one consisting two clusters, including 14 *sca*A (grey) and 12 *sca*C (ATD in C,D has an additional *sca*: UT144) (pink), and another one consisting of four clusters, including 4 *sca*B (blue), 12 *sca*D (green), 12 *sca*E (purple) and 4 *sca*F (red). Neighbor-Joining trees are shown in S1 Fig.

Multiple sequence alignments for the SP-PD, and ATD *sca* nucleotide and Sca protein sequences were performed using MUSCLE [34]; subsequently, the phylogenetic trees were constructed using Maximum Likelihood (ML), and Neighbor Joining (NJ) approaches. Model JTT+G+I/GTR+G and JTT+G/GTR+G+I (JTT: Jones-Taylor-Thornton; GTR: General Times Reversible; I: Invariable sites, G: rate variation among sites, I+G: both) were identified as the best substitution models by the Model Test program for reconstructing the ML tree in MEGA7 with 100 bootstrap supports for SP-PD and ATD domain protein/gene sequences, respectively [36] (Fig 1). The NJ tree was constructed under *p-distance* [37] with 100 bootstrap supports (S1 Fig).

### Primers and probes; TaqMan assay design

**Fig 2:**
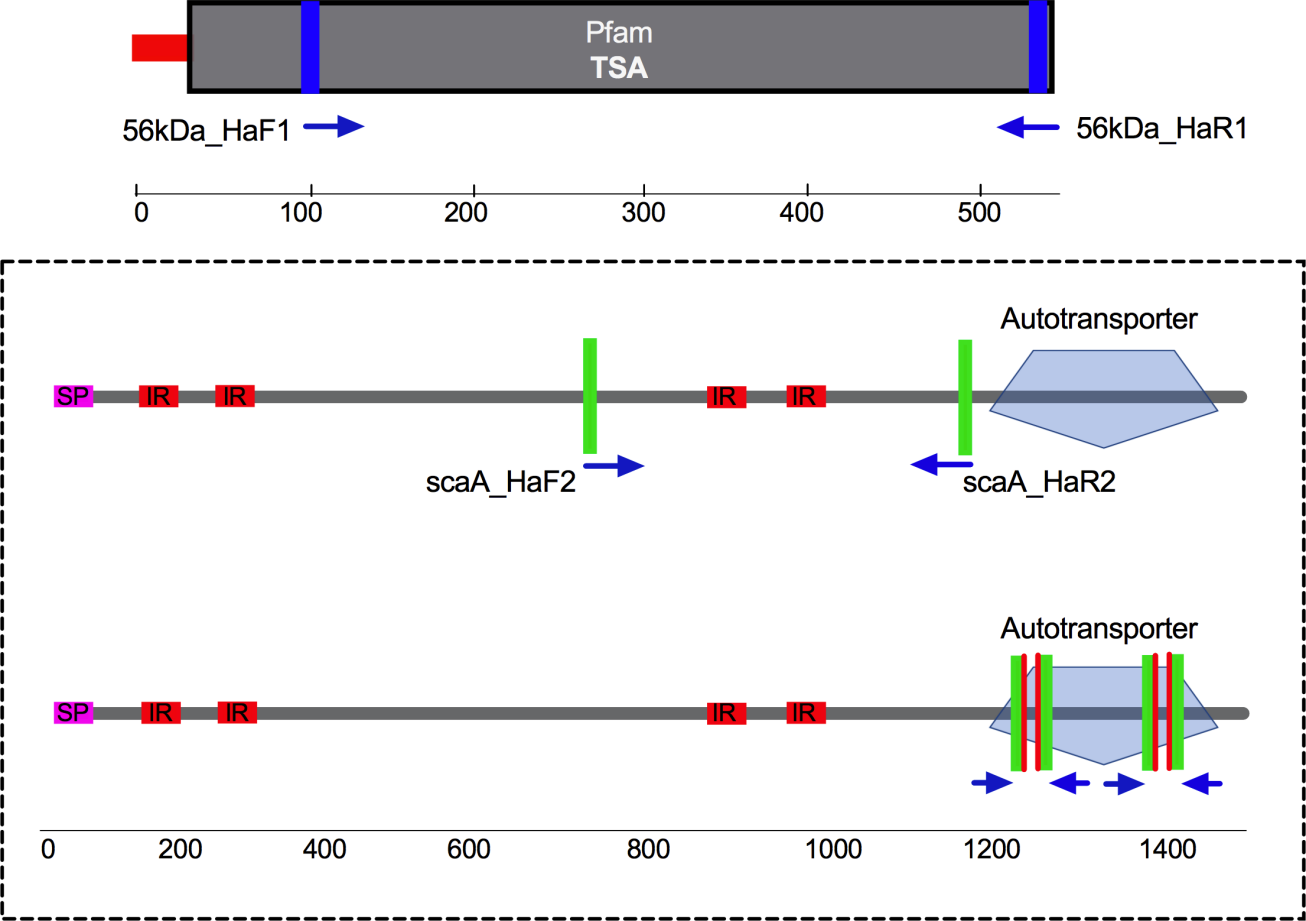
ScaA Boryong primer sites and ATD TaqMan assay design. Top: Ha (2012) conventional primers for 56 kDa antigen gene (TSA) detection. Middle: Ha (2012) conventional PD primers for *sca*A detection. Bottom Two *sca*A ATD sites with conserved primer sequences; each site was evaluated with TaqMan probes binding to each strand of the amplicon.

**Fig 3:**
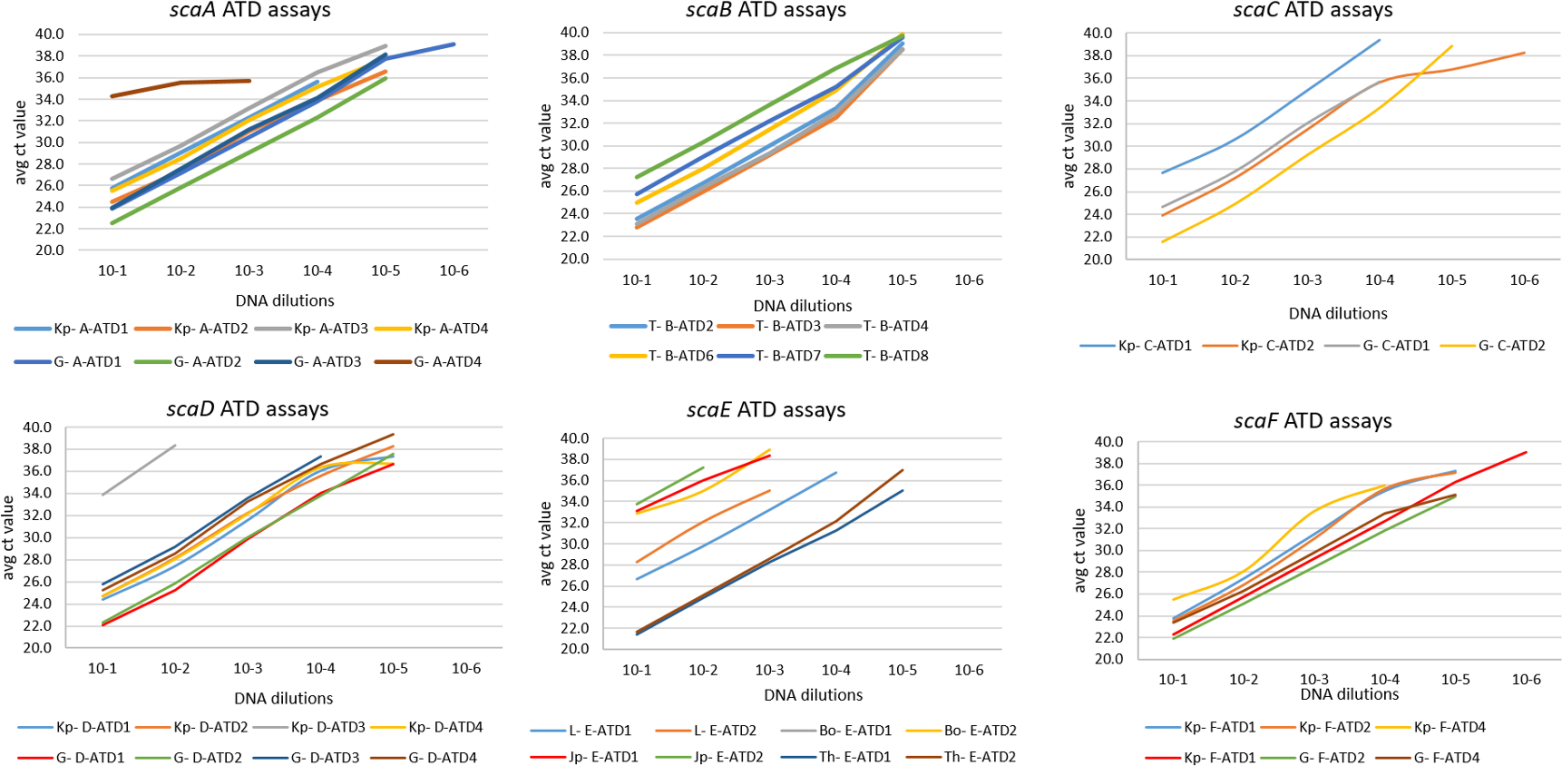
Comparison of TaqMan assays for efficiency of detection of *sca*A-*sca*F ATD amplicons. For each panel the specific primer and probe combinations used for the DNA dilution series are as indicated in S3B Table, S3C Table. Kp-Karp DNA, G = Gilliam DNA, T = TA716 PP DNA, L = Levis DNA, Bo = Boryong DNA, Jp = 34483 (Japan3) DNA, Th = TH1813 DNA.

**Fig 4:**
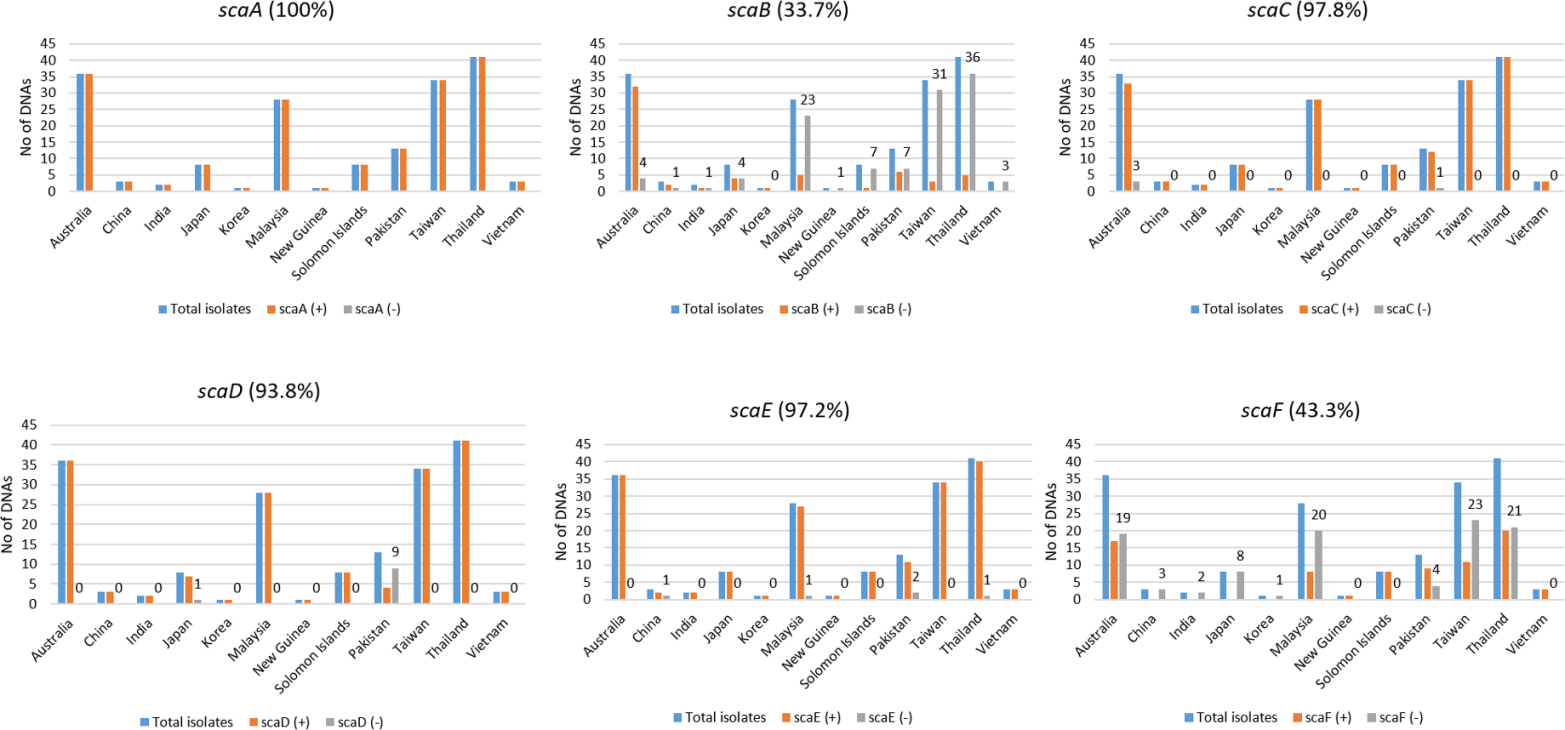
TaqMan ATD survey of *sca*A to *sca*F genes in Ots isolates from different countries. For each panel the best selected primer and probe combinations used are, *sca*A (A-ATD2), *sca*B (B-ATD3 and B-ATD6), *sca*C (C-ATD2), *sca*D (D-ATD1 and D-ATD2), *sca*E (E-ATD1 and E-ATD3), *sca*F (F-ATD2). The primer and probe combinations used are as indicated in S3B Table, S3C Table. Percentages are summarized in S3D Table.

Tandem repeats (TR) in the *sca* genes were identified using the Tandem Repeat Finder program (http://tandem.bu.edu/trf/trf.basic.submit.html) to avoid including those repeated regions when primers and probes were designed [38]. None of the large tandem repeats were in the *sca* ATD. The ATD was highly conserved between the DNAs of different genotypes compared to the *sca* passenger domains (Fig 1, S1 Fig, S2 Table) but each Taqman assay (*sca*A-*sca*F) was *sca* specific by BLAST analysis of the primers and probes and differences in amplicon sizes and reactions with different DNAs (S1 Table). Multiple primers and probes were designed for conserved regions in the flanking 5’ and 3’ regions, SP-PD and ATD of *sca*A, *sca*B, *sca*C, *sca*D, *sca*E and newly identified *sca*F (S3 Table, Figs 2 and 3). The best primer pairs were selected by conventional PCR with prototype control *Orientia* DNAs (KarpPP, Boryong B, KatoPP, Gilliam PP, AFSC7, Calcutta) to identify those providing good amplification of *sca* gene regions without spurious bands, with minimal primer-dimer formation, and of the expected amplicon size. Control DNAs were serially diluted from 10–1 to 10–6 dilutions from working stock and the sensitivity of different probe/primer combinations were evaluated by performing TaqMan assays on control *Orientia* DNAs as described below (Fig 3). Optimal TaqMan primer and probe combinations were then selected for the *sca* gene survey of the Ots isolate DNAs (Fig 4).

### *Orientia tsutsugamushi* DNAs

Gamma irradiated or cycloheximide treated (0.5 μg/ml) L929 cell passaged well-characterized reference isolates (CDC collection) of *Orientia tsutsugamushi* from 12 different countries (Table 1) were extracted with single Qiagen spin columns as described previously [39]: Medium and cells harvested from one T150 flask with glass beads was centrifuged at 8000 rpm for 10 minutes and then washed in 1 ml of phosphate buffered saline; one half of the pellet was lysed and purified with the Qiagen spin column; the column DNA was then eluted into 0.5 ml of Qiagen AE buffer and stored at 4°C [38]. Working stocks (1:10 dilution in AE) of these master stocks of Ots DNAs were used for all molecular analyses.

**Table 1:** Countries of origin of *Orientia tsutsugamushi* isolate DNAs tested.

Country	Number of isolates	Percentage of isolates
Australia	36	20.2
China	3	1.7
India	2	1.1
Japan	8	4.5
Korea	1	0.6
Malaysia	28	15.7
New Guinea	1	0.6
New Zealand	8	4.5
Pakistan	13	7.3
Taiwan	34	19.1
Thailand	41	23.0
Vietnam	3	1.7
Total	178	100.0

### Conventional PCR analysis

Conventional PCR was done in an Eppendorf master gradient cycler in a total reaction volume of 20 μl. The mixture consisted of 2 μl of DNA working stock template, 10 μl of 2x concentrated Qiagen PCR master mix, 0.5 μl (20 μM) each of both forward and reverse primers and 7 μl of molecular grade water. The cycling conditions were initial denaturation at 95°C for 5 minutes followed by denaturation at 95°C for 1 minute, annealing at 57°C for 2 minutes and extension at 70°C for 2 minutes. The above cycle was repeated for 35 times followed by a final extension step at 70°C for 2 minutes. The PCR products were run on 1–3% agarose gels in Tris-borate buffer at 80 V based on the anticipated length of the PCR amplicon along with molecular markers (BioRad Laboratories EZ load 100 bp or 1 KB plus molecular rulers). Documentation of ethidium bromide stained gels post electrophoresis was done on a BioRad Gel Doc imager and stored for further size analysis.

### *Sca* autotransporter domain TaqMan real time qPCR assays

The *sca* ATD assays were conducted in a total volume of 20 μl of qPCR mixture in 96 well plates. The mixture consisted of 2 μl of DNA working stock template or no template control added to a master mix comprised of 10 μl of 2x concentrated iTaq universal probes super mix (Bio-Rad), 6.8 μl H_2_O, 0.5 μl (20 μM) of both forward and reverse primers and 0.2 μl (10 μM) of each probe (all 5’-FAM, 3’ BHQ1 labeled) for each of the *sca* targets. The cycling conditions were initial denaturation at 95°C for 3 minutes followed by 40 cycles of two step amplification: denaturation at 94°C for 15 seconds and 60°C for 60 seconds on a Bio-Rad CFX 96 thermocycler. Plate reading for fluorescence was recorded during every thermal cycle at the annealing step and data was analyzed using Bio-Rad CFX software manager version 3.0. To enable accurate direct comparison of the different primer probe combinations evaluated for each *sca* ATD target assay, they were all analyzed on the same microplate with aliquots of the same DNA dilution series.

### Construction of ATD domain plasmids and specificity testing of ATD TaqMan assays

Recombinant pCR 2.1 plasmids carrying the ATD regions of each of the 6 sca’s were generated following the TA cloning strategy (In Vitrogen, ThermoFisher Catalog K202020). Briefly, each of the *sca* ATD regions (S3D Table) were PCR amplified and checked for amplicon purity on 2% agarose gels. Amplicons ligated into pCR 2.1 were transformed into One Shot Competent Cells (TOP 10) and screened for transformants by plating on LB agar plates with 50μg/mL of kanamycin, overlaid with 40μl of 40mg /mL X-Gal. Plates were Incubated overnight at 37ºC. Four well-spaced white colonies from each transformation plate were selected and colony PCR was performed to confirm successful transformation and size of the insert. Positive clones by colony PCR were cultured in 5 mL of 50μg/mL of kanamycin overnight and plasmids were purified using Qiagen plasmid mini kit. For *sca* qPCR specificity experiments, plasmid DNA quality and yield (Qubit Double Strand DNA assay) were determined and equal amounts of two dilutions of each plasmid were added as DNA templates for TaqMan reactions (S4 Table). Each of the 6 plasmids (two different amounts) were screened with all of the *sca* primer and probe combinations (9 ATD assays) along with positive *Orientia* DNA and no template controls.

## Results

### Characteristics of *sca* gene sequences in *Orientia* genome sequences

Six Sca gene proteins were detected by tBLASTn in the 14 *Orientia* genome sequences available (S1 Table, Fig 1). The gene clusters of the maximum likelihood (ML) and Neighbor Joining (NJ) phylogenetic trees matched those expected from the Boryong ScaA-E and Karp ScaF tBLASTn predictions (Fig 1, S1 Fig). The new type *sca*F was only found in three isolates (Karp, AFSC7, Sido) including both the independently obtained Karp2M and KarpIGS sequences. *Sca*B was also only present in 3 isolates but 2 identical copies were detected in the Boryong sequence. *sca*A, *sca*C, *sca*D, and *sca*E were present in all genomes except Sido which had the least genome coverage. However, except for Boryong and Ikeda, the other genome assemblies are incomplete and comprised of many contigs; consequently, it is not possible to know whether the *sca*B and *sca*F genes are really absent or just not found in the available incomplete assemblies. Several probable frame shift errors and truncations of *sca* protein genes were found and the reading frame (RF) corrected sequences are shown in S1 Table. *sca*B size was highly conserved with only insignificant size differences found among the two 1950 bp Boryong copies, and the TA716 (1995 bp) and Sido (1998 bp) sequences. The three *sca*F gene sequences were nearly identical in sequence and were identical in size (1938 bp). Among the other four *sca* genes, the *sca*C genes had the least size variation, varying between 1554 bp (UT76) and 1581 bp (Boryong) and contained two tandem repeats accounting for most of the small size differences. The *sca*D gene sizes were quite variable from 1998 bp (Kato) to 2997 bp (Karp) due in part to varying numbers of tandem repeats and some large INDELs. The *sca*A genes were the largest (4344–4722 bp) and varied in tandem repeat structure, length of an encoded polyQ region, and the type of INDELs present. UT76 had two copies of *sca*A with slightly different sizes, with different tandem repeats and sequence divergence. The *sca*E genes exhibited moderate size differences between 2238 bp (AFSC4) and 2283 (Boryong). The SP-PD domains were significantly more divergent than the ATD beta barrel for each *sca* gene (Fig 1, S1 Fig, S2 Table). Consequently, we decided to evaluate the conserved sites in sequence alignments of SP-PD and particularly, the ATD regions to develop sensitive surveillance methods for the *sca* genes in our collection of isolates of Ots.

### Phylogenetic analyses of Sca gene sequences

All four ML trees (amino acid and nucleotide of SP-PD and ATD domains) indicated the presence of two major *sca* gene clades, one consisting of two clusters (class-1), including *sca*A and *sca*C and another one consisting of four clusters (class-2), including *sca*B, *sca*D, *sca*E and *sca*F with 90–100% bootstrap support in the majority of the major nodes (Fig 1). Within each *sca* gene clade, the isolate branchings were similar for *sca* types with more sequences (*sca*A, *sca*C, *sca*D, *sca*E) and the same pattern was generally seen with the NJ trees as found by ML (S1 Fig). This indicates that the two classes of *sca* were closely related and likely diverging from a common ancestor. The overall topologies of ML and NJ trees were quite similar, however, some minor differences in branching order within the clusters and cluster arrangements, particularly in class-2 *sca* types were apparent.

### *Orientia tsutsugamushi* DNAs tested

Overall, 181 non duplicate DNA samples were evaluated in the present study; 178 were from reference isolates of Ots and 3 were from uninfected cell cultures. These 3 uninfected DNAs served as negative controls in all the experiments. The 178 isolate DNAs originated from 12 different countries (Table 1) These isolates are all well characterized CDC reference strains which all grow and stain like *Orientia* and all contained the *Orientia* scaA gene (Table 2).

**Table 2:** Efficiency of conventional and *sca* ATD TaqMan assays for detecting *sca* genes in Ots isolates.

Target (primers)	Number positive (178 isolate DNAs)	Amplicon size in bp	Percentage positive
56kDa TSA c-PCR (HA primers) [28]	106	1344	59.6
*sca*A c-PCR (Ha et al. primers) [28]	111	1121	62.4
*sca*A c-PCR (3811/3815)	175	586	98.3
*sca*A ATD-TaqMan assay A-ATD2 (3811/3812)	178	139	100.0
*sca*B ATD-TaqMan assay B-ATD3 (7197/7193)	60	141	33.7
*sca*C ATD-TaqMan assay C-ATD2 (6979/6985)	174	145	97.8
*sca*D ATD-TaqMan assay D-ATD1 (7023/7030)	167	138	93.8
*sca*E ATD-TaqMan assay E-ATD3 (7125/7131)	173	134	97.2
*sca*F ATD-TaqMan assay F-ATD2 (7172/7173)	77	154	43.3

### Detection of *Orientia tsutsugamushi sca*A by conventional PCR

The previously published 56 kDa TSA primers of Ha et al [28] amplified a 1344 bp product and Ha *sca*A passenger domain primers amplified an 1121 bp product (Fig 2). The Ha TSA and scaA primers were used as DNA quality controls to evaluate whether primers derived from more conserved sites on the *sca*A gene ATD alignment would be more efficient in detecting this gene (S3 Table, Fig 1D). After screening multiple *sca*A primers by PCR with DNAs from KarpPP, KatoPP and GilliamPP prototypes, the best primer pair that amplified a *sca*A product (586 bp) with a good intensity and without spurious bands was selected to screen all the DNAs (Table 2). The 56kDa Ha primers detected 106/178 DNAs (59.6% positivity) and *sca*A Ha PD primers identified *sca*A in 111/178 DNA’s (62.4% positivity). Our conventional *sca*A ATD PCR primers were much more sensitive in that they amplified *sca*A from 175 of 178 Ots DNAs with high sensitivity (98.3%). This confirmed that the quality of the Ots DNAs we used was excellent and that these *sca*A gene ATD sites were indeed sufficiently conserved across a wide range of TSA genotypes of Ots isolates to be useful.

### ATD TaqMan survey of *Orientia tsutsugamushi* DNAs for *sca*A*—sca*F genes

To increase the likelihood of detection of *sca* genes with divergent passenger domain sequences (using the sequence alignments for all of the available *sca* genes and proteins), we selected unique conserved regions of the ATD domains for each of *sca*A-*sca*F genes (Fig 1, S1 Fig, S2 Table, S3 Table) to develop ATD TaqMan assays. Four such TaqMan assays were tested (two sites each with two probes each) (Fig 2 general design, S3 Table) for linearity and sensitivity of detection with serial tenfold dilutions of prototype Ots DNAs (Fig 3). We evaluated the *sca* specificity of each assay (lack of cross-over between *sca* targets) by testing the best assay against cloned ATD target plasmids for each *sca* gene (S4 Table). None of the assays showed cross-talk between *sca* genes and they all showed similar levels of sensitivity for the same control DNA and each of the *sca* plasmids. That assay for each target *sca* ATD was then used to survey all the Ots isolate DNAs in Table 1 for the presence of each *sca* gene (Table 2, Fig 4).

Among the 178 Ots DNAs tested with the six ATD TaqMan assays, all the isolate DNAs tested were positive for *sca*A gene (Table 2) while the 3 control DNAs gave no signal. The *sca*C, *sca*D, and *sca*E ATD targets were also detected with very high prevalence rates (Table 2, Fig 4). Consistent with the available Ots genome sequence data, *scaB* and *scaF* were less prevalent but they were both detected at higher rates than the genome sequence data would suggest. This result could be due to the bias in the sequence data inherent in being derived from a high proportion of strains from Thailand. To examine this possibility, we also partitioned the prevalence of *sca* genes by country (S3D Table, Fig 4). The *scaA* ATD target was detected in all the 178 isolates tested. The *sca*B ATD target was detected in the least number of isolates tested. Except for the one isolate from Korea, which was positive, there were isolates negative for *sca*B from every other country. However, most of the Australian isolates (88.9%) were positive for *sca*B. The *sca*C ATD was detected in 97.8% of the DNA’s tested. Only 3 isolates from Australia and an isolate from Pakistan were *sca*C negative. The *sca*D ATD target was detected in 93.8% of the DNAs tested. An isolate each from Japan and many isolates from Pakistan (9 isolates) were negative for *sca*D. The *sca*E ATD target was detected in 97.2% of the DNAs tested. A single isolate from China, Malaysia, Thailand and 3 isolates from Pakistan were negative for *sca*E. The *sca*F ATD target was detected in 43.3% of the DNAs tested and with all of the Solomon Islands and Vietnam DNAs positive. However, more than half (51.2–71.4%) of the isolate DNAs tested from Australia, Malaysia, Taiwan and Thailand were negative for *sca*F.

## Discussion

The immunodominant major TSA of Ots is the most extensively studied serological and molecular target in scrub typhus diagnosis; however, it exhibits great antigenic diversity in each of its four variable domains [29]. Similarly, AT proteins comprise one of the largest and functionally diverse group of secreted and outer membrane proteins found in GNB and play an important role in their virulence [13, 40]. From our work at least six *sca* AT paralogs are now known to be present in Ots and four of those are widely distributed throughout the endemic region for scrub typhus. *sca*A functions as an adhesion factor in Ots and anti *sca*A antibody significantly neutralized Ots infection of host cells [24, 27]. Additionally, immunity to heterologous strains was observed for Ots when vaccination was performed with ScaA combined with 56kDa STA [27]. ScaA bound to zinc oxide nanoparticles also provided good homologous protective immunity [41]. Previous immunological work on Sca proteins used the Boryong genotype of Ots, the predominant endemic strain causing scrub typhus in Korea [42] but it is not the predominant strain in other endemic countries [43–46]. Indeed, the Boryong Sca genes were outliers phylogenetically so the probable immunological properties of other Sca proteins in other Ots isolates needs to be confirmed. Indeed, the presence and absence of antigenically different Sca proteins in different Ots isolates (S3D Table) may account for some of the clinical and epidemiological strain specific differences seen with this species. [29, 42].

Using the previously published conventional PCR primers that amplified 56kDa TSA and *sca*A genes, [28], the lower percentage positivity for these genes was 106 (59.6%) and 111 (62.4%) of the Ots isolates, respectively. The significant proportion of DNAs negative for these PCR’s is likely due to the primer design because these researchers used the outlier Boryong *sca* sequences for their primer design. The demonstrated benefit of using conserved regions of more DNA sequences for scaA for both conventional PCR and TaqMan ATD assays allowed us to design highly effective assays for three of the other *sca* genes as well. However, the *sca*B and *sca*F primer designs are limited by the same lack of sequences faced by Ha et al. [28]. Since we have identified additional isolates with different sequences of these genes, we expect those assays can be improved to be more efficient with better primer selection. On the other hand, the sensitivity and efficiency of the other four *sca* ATD TaqMan assays (*sca*A, *sca*C, *sca*D, *sca*E) appear to approach that of other quantitative PCR assays for *Orientia* that are routinely used for detection in clinical, animal, and chigger samples based on our analysis of these same DNAs with other Ots TaqMan assays [39].

*Sca*A is the largest of the Ots *sca*’s and appears to be universally distributed based on our survey. This suggests it has an essential role in the pathogenesis of this agent but the extent to which its variability affects the clinical manifestations of different Ots isolates is unknown. *Sca*B seems to be the outlier among all the *sca*’s as it was detected in the smallest number of the strains tested despite being present in two copies in Boryong isolate. Its biological role and immunogenicity has not been studied. The *sca*C gene is the smallest Ots *sca* and is most conserved in size based on available the *sca*C sequences (S1 Table). Owing to these results, *sca*C seem to be a suitable candidate either individually or along with *sca*A for use in serodiagnostic assays and for evaluation as a vaccine candidate. Whether the apparent large size differences in *sca*D are due mostly to variable passenger domain tandem repeat differences and whether those will affect the biological properties and immunogenicity of Ots isolates is also unclear. The medium size *sca*E gene is also well conserved but this increase in size over *sca*C may make it more difficult to clone and purify for use in serodiagnostic assays and vaccines. The newly identified *sca*F was found in only 43.3% of the tested isolates but, as noted above, this may be an artefact of the limited number of sequences available for primer design of the ATD-TaqMan assay.

The percent identity matrix [47] for the complete proteins of the six *sca* types exhibited substantial intra cluster variability in both class-1 (*sca*A, 73.29–100%; *sca*C, 86.85–100%) and class-2 (*sca*B, 55.99–100%; *sca*D, 76.04–100%; *sca*E, 74.49–100%; *sca*F, 99.53–100%) *sca* types. How much this is biased by the limited number of sequences available at this point, remains to be determined so it is probably quite premature to try to assess whether there are stronger evolutionary pressures on any particular Sca protein category or either class of Sca. As the number of cases and outbreaks of scrub typhus has been continuously increasing in recent years, improved diagnostic assays based on the *Orientia sca* autotransporter proteins may contribute to earlier and more accurate diagnosis of scrub typhus. However, their primary importance may lie in the largely unexplored realm of their interactions with the host cells that are invaded by *Orientia* during scrub typhus infections. Whether they will be suitable vaccine candidates that can enhance TSA mediated immunity is also an important issue to resolve.

In summary, we have shown that there is genetic diversity in the sequences and distribution of different *sca* genes among diverse isolates of *Orientia tsutsugamushi* and that these six paralogous AT genes have evolved independently, probably after early gene duplication events much as was detected for *sca*B in Boryong isolate. It is possible recombination between these proteins may occur but branching topology of each gene family does not yet support that possibility. As more genome sequences of diverse Ots isolates become available, it is quite possible that additional paralogous Ots *sca* genes may be identified that could rival the complexity of the *sca* gene family found in species of *Rickettsia*.

## Supporting information

S1 Fig Phylogenetic relationship of *sca* sequences (types A-F) of *O*. *tsutsugamushi*. The Neighbor-Joining trees show the phylogenetic relationship of the known *sca* protein genes, based on SP-PD region A) protein and B) gene sequences and ATD region C) protein and D) gene sequences. Bootstrap values (percentages of 100 replications) above 60% are indicated at the nodes. All four trees show two major clades, one consisting of two clusters, including 14 *sca*A (grey) and 12 *sca*C (ATD in C,D has an additional *sca*: UT144) (pink), and another one consisting of four clusters, including 4 *sca*B (blue), 12 *sca*D (green), 12 *sca*E (purple) and 4 *sca*F (red).
(TIF)

S1 Table Sca Protein Genes in *Orientia* Isolates. The Sca protein gene sequences identified by tBLASTn with known Boryong (ScaA-E) or Karp (ScaF) sequences and their coordinates and sizes in Ots NCBI contigs are shown. The closest homolog identified (S2 Table) is also noted Sequences obtained by Ha et al. (2012), the sources of the partial sequence (p) corrected sequences (reading frame-RF shift issues-cor) used for Fig 1 and Supplemental Fig 1 are also shown.
(XLSX)

S2 Table Amino acid identity and percentage distance between Ots Sca protein domains. Different tabs show the Muscle alignment calculations from Geneious for the number of identical amino acids (top triangle in blue) and %amino acid similarity (bottom triangle in black) for each of the six Sca protein alignments. The SP-PD data is in the top of each table and the ATD data at the bottom of each table.
(XLSX)

S3 Table Primers and Probes for *Orientia* Sca protein genes. Tabs: Table 3A. c-PCR primers for Fig 3; Table 3B. *sca* ATD primers, probes; Table 3C. ATD TaqMan Assays; Table 3D. Country *sca* survey data.
(XLSX)

S4 Table Specificity of *sca* Autotransporter Domain TaqMan Assays. (XLSX)
