# Supplementary figures and images for: Detection and distribution of Sca autotransporter protein antigens in diverse isolates of Orientia tsutsugamushi
Source: PLoS Negl Trop Dis. 2018 Sep 20;12(9):e0006784. doi: 10.1371/journal.pntd.0006784 (PMC6168176; doi:10.1371/journal.pntd.0006784)

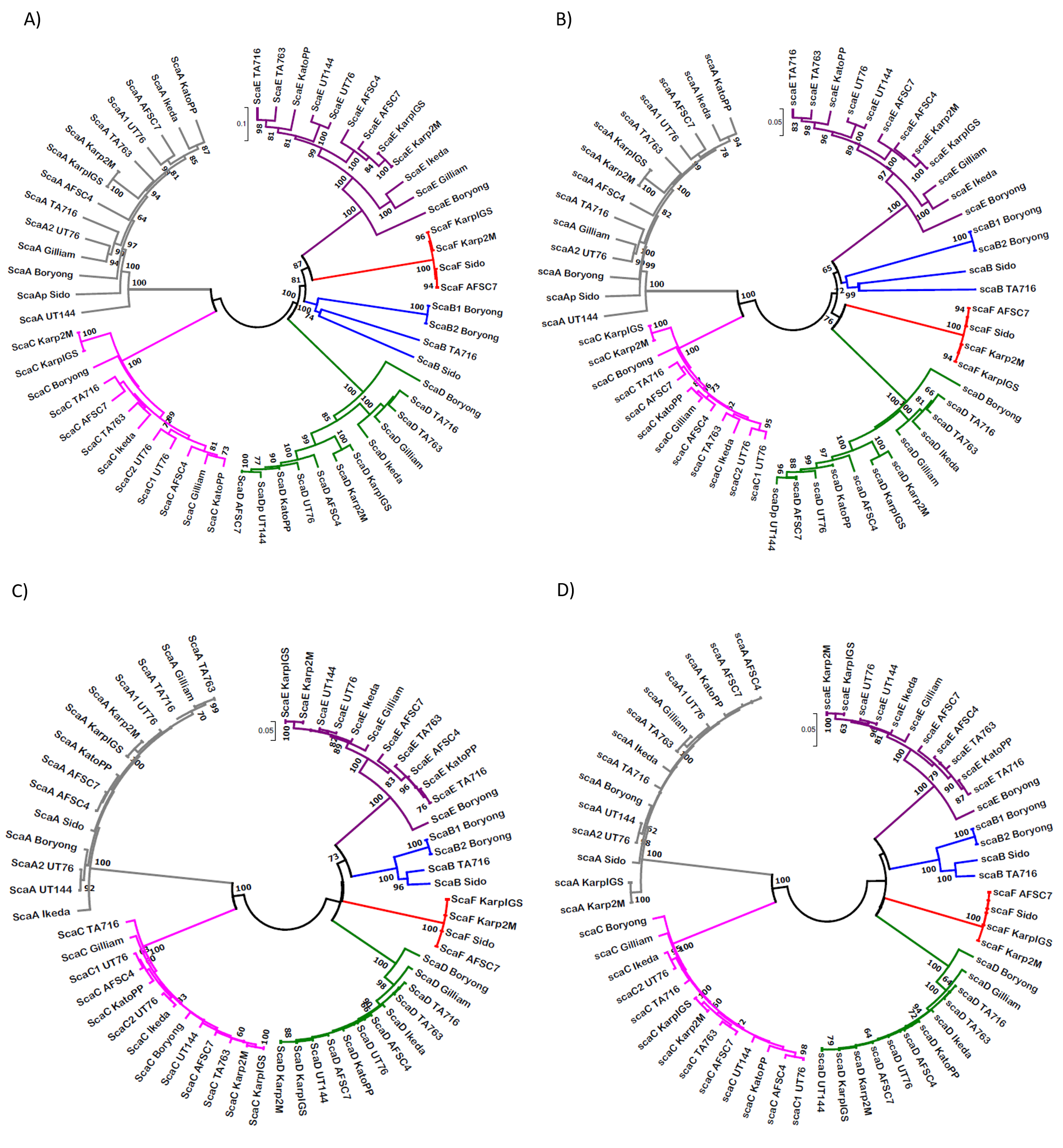

Supplement: S1 Fig — The Neighbor-Joining trees show the phylogenetic relationship of the known sca protein genes, based on SP-PD region A) protein and B) gene sequences and ATD region C) protein and D) gene sequences. Bootstrap values (percentages of 100 replications) above 60% are indicated at the nodes. All four trees show two major clades, one consisting of two clusters, including 14 scaA (grey) and 12 scaC (ATD in C,D has an additional sca: UT144) (pink), and another one consisting of four clusters, including 4 scaB (blue), 12 scaD (green), 12 scaE (purple) and 4 scaF (red). (TIF) [file pntd.0006784.s001.tif]
